# Evaluation of canine tympanic membrane integrity using positive contrast computed tomography canalography

**DOI:** 10.3389/fvets.2024.1304066

**Published:** 2024-07-12

**Authors:** V. Alves-Nores, M. J. Plested, R. Rubial, R. Salgüero

**Affiliations:** ^1^Diagnostic Imaging Department, Lumbry Park Veterinary Specialists, Alton, United Kingdom; ^2^Hospital Veterinario Puchol, Madrid, Spain; ^3^AniCura Imavet Referencia Veterinaria, Santiago de Compostela, Spain; ^4^Veterios Hospital Veterinario, Madrid, Spain; ^5^Diagnostic imaging department, University Complutense of Madrid, Madrid, Spain; ^6^Vet Oracle Teleradiology, Diss, United Kingdom

**Keywords:** CT canalography, contrast study, tympanic membrane, dogs, otitis media (OM)

## Abstract

**Introduction:**

The assessment of the integrity of the tympanic membrane (TM) can be a diagnostic challenge in patients with otitis externa and/or otitis media (OM) under an otoscopic examination. In computed tomography (CT), the TM is not always visualized. However, a positive contrast canalography using conventional radiography has been previously described to evaluate the TM integrity.

**Objective:**

This single-center study aimed to describe the positive contrast canalography technique in CT and its usefulness in identifying TM integrity in 11 dogs.

**Methods:**

Dogs with clinical signs of ear disease underwent CT canalography following a systematic protocol.

**Result:**

The presence of contrast medium and contrast homogeneity within the tympanic cavity was assessed, confirming TM rupture when contrast medium was present in the tympanic cavity. CT canalography was performed in 16 ears, and in 7 of the 16 ears (44%), there was a contrast in the tympanic cavity, confirming TM rupture (positive CT canalography result). In two of the seven cases (29%), rupture of the TM was identified in both otoscopic and CT canalography. In the remaining five of the seven (71%) positive CT canalography results, tympanic membrane rupture was identified only by CT canalography.

**Discussion:**

In conclusion, a positive contrast CT canalography is a complementary and safe technique to assess TM integrity, with a potentially higher sensitivity than otoscopic examination for the diagnosis of TM rupture in dogs.

## 1 Introduction

In dogs, otitis media (OM) is often caused by an extension of and/or failure in treating external ear disease (1). In veterinary medicine, the visualization of the tympanic membrane (TM) and the diagnosis of OM during a conscious otoscopic examination can be challenging due to the long, curved, and funnel-shaped conformation of the ear canal of a healthy mesaticephalic dog. In addition, the integrity of TM can be almost impossible to assess in patients with chronic external and/or middle ear disease (2). Furthermore, external ear canal stenosis manifests commonly in instances of chronic otitis externa or may be attributable to natural breed variations, particularly observed in brachycephalic breeds (3, 4), with or without variable volumes of intraluminal secretions, making the examination more complicated. Currently, the gold standard technique to assess the TM is video-otoscopy (5).

The absence of the visualization of the TM during an otoscopic examination can suggest the presence of an underlying OM (6). It is important to identify the integrity of the TM before either selecting a suitable medical treatment option, as some topical products can be toxic to the inner ear, or planning a surgical approach in cases of external and middle ear disease (7).

Advanced imaging techniques such as computed tomography (CT) and magnetic resonance imaging (MRI) are commonly used to evaluate the ears in veterinary patients. CT is generally more used in practice as it is more accessible, cheaper, provides an excellent resolution of the bony structures, and requires a shorter time under general anesthesia. Hence, it is considered the gold standard imaging modality to assess the middle and inner ear in dogs and cats (7–9). The visualization of the TM in CT can identify the TM in some but not all dogs. The best plane reported for the visualization of the TM is the dorsal plane; however, its visualization strongly depends on the window selection (10).

Positive contrast canalography and subsequent radiography have been previously described in the veterinary literature as a valid method for the evaluation of the external ear canal diameter and for the detection of a possible rupture of the TM (11, 12). This prospective study aimed to (i) describe the procedure of a CT canalography technique in dogs with external and/or middle ear disease and (ii) describe the usefulness of CT canalography in identifying rupture of the TM in dogs.

## 2 Materials and methods

This study was conducted between January 2019 and June 2020 at the referral center Hospital Veterinario Puchol in Madrid, Spain. The inclusion criteria for this study were dogs with a clinical presentation of unilateral or bilateral external and/or middle ear disease referred with poor response to medical therapy. Appropriate clinical consent, outlining the relevant risks and complications, was obtained from all owners.

All dogs underwent a physical clinical examination, complete blood count (CBC), and biochemistry, and a conscious conventional otoscopic examination was performed with an otoscope and an otoscope speculum before the CT examination by a clinician. Sampling for microbiological culture and cytology was performed as required. Each ear was inspected otoscopically to assess the external ear canal, the intraluminal content, the visualization of the tympanic membrane (yes or no), and in cases where the TM was visible, whether there was evidence of rupture (yes or no). The video-otoscope examination was conducted under general anesthesia after the CT examination.

### 2.1 Positive contrast CT canalography protocol

In each patient, a CT scan of the head was performed using a *16-slice multidetector helical CT (Aquilion Lightning, Canon Medical Systems, Madrid, Spain)*. All patients were under general anesthesia and positioned in sternal recumbency. The scan length extended from the nares to C2, including the retropharyngeal lymph nodes. A helical scan protocol, from rostral to caudal, was used with a 0.5-mm slice thickness, a pitch of 0.5, and a 512 × 512 matrix. The scan utilized a transverse plane soft tissue and bone reconstruction algorithms with a 1-mm and 0.5-mm slide thickness, respectively, in the pre-contrast study. Subsequently, 2 mL/kg of iodinated non-ionic contrast medium (*Optiray^®^, ioversol 300 mg/mL*) was administered manually via a cephalic vein. A post-contrast CT was performed with the same parameters, using a soft tissue reconstruction algorithm with the same slide thickness.

**Figure 1:**
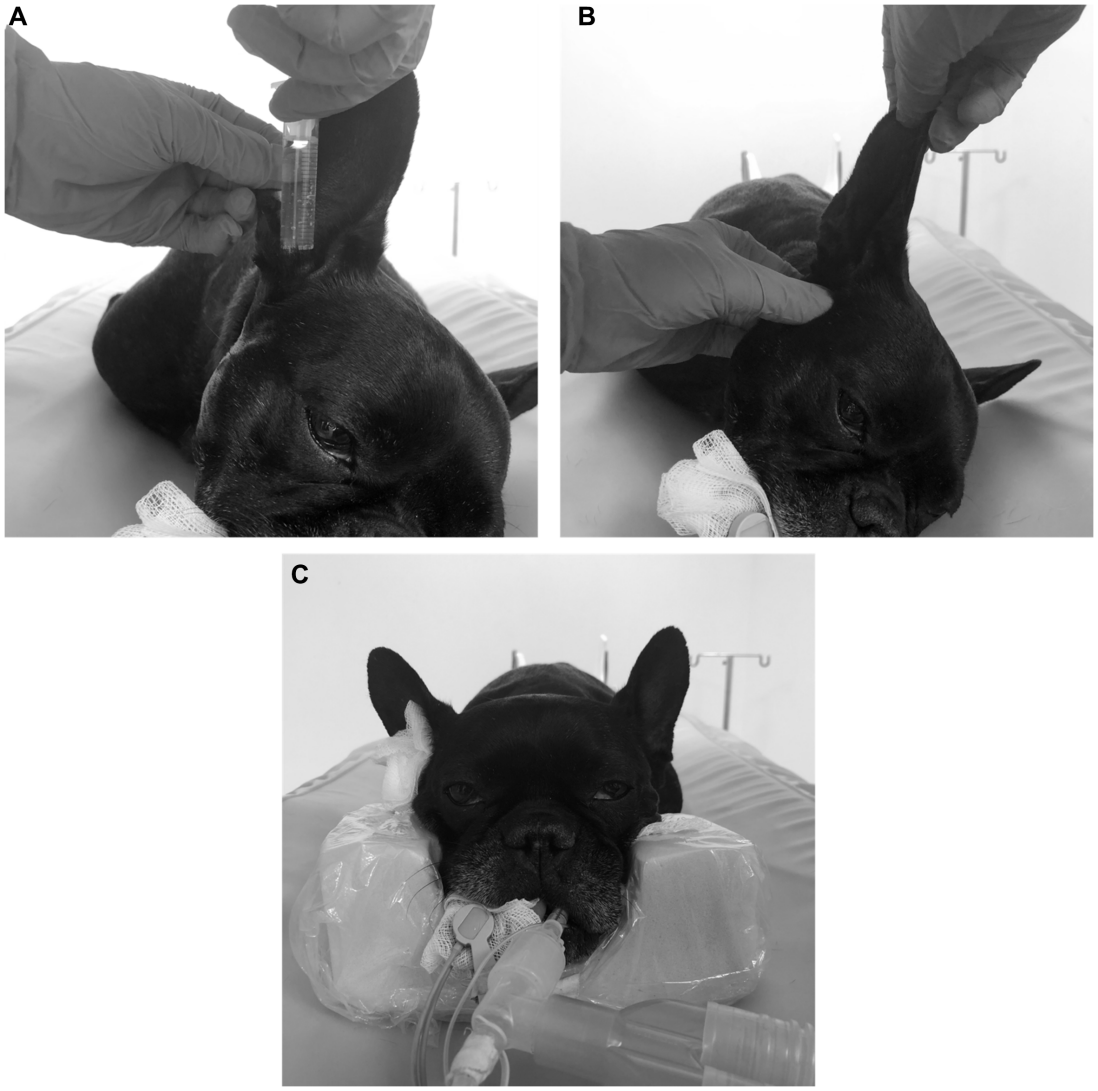
Positive contrast CT canalography dilution injection technique description. **(A)** The head positioned in lateral recumbency with the clinically affected ear canal situated uppermost and the non-ionic iodinated contrast dilution injected into the external ear canal. **(B)** The ear canal massaged gently for 30 s to distribute the contrast medium within the ear canal. **(C)** A cotton wool ball placed in the outer part of the external ear canal to plug the ear canal. Afterward, the patient was repositioned in sternal recumbency.

In addition, a CT canalography was performed in those ears with aural clinical signs, using the following systematic approach: the head was positioned in lateral recumbency with the affected ear canal positioned uppermost. The ear canal was flushed with sterile saline and then cleaned with a cotton swab prior to the topical contrast administration. A volume of 2.5 mL of non-ionic iodinated contrast medium was mixed with an equal volume of sterile saline, obtaining a dilution of 1:1. The external ear canal was filled up with the contrast solution using an intravenous catheter (*Braum^®^ IV catheter, 20G x 2″*) (Figure 1A), and half of the catheter was introduced into the canal. Then, the external ear canal was massaged gently for 30 s to distribute the contrast medium within the ear canal (Figure 1B). A cotton wool ball or a standard swab was placed in the outer part of the vertical external ear canal, intentionally positioned outside the canal’s lumen to prevent the absorption and potential leakage of contrast material. Subsequently, the patient was repositioned in sternal recumbency (Figure 1C). A “limited bulla-CT” scan protocol was then performed, utilizing the same technique as the one mentioned above, but scanning only the rostrocaudal length of the ears, using bone and soft tissue algorithms with 0.5 and 1 mm slice thickness. If the other ear also needed to be assessed, the same protocol was repeated, including the “limited bulla-CT” of the second ear. At the end of the studies, the ear/s were flushed and cleaned again with saline to remove all the contrast medium administered.

### 2.2 CT imaging features

All the images were assessed with multiplanar reconstruction in both soft tissue (WL: 50, WW: 350) and bone (WL: 555, WW: 2550) reconstruction windows, using a DICOM viewer [*OsiriX MD Dicom Viewer Pixmeo Sarl^®^, version 13.0.1, Geneva, Switzerland* and *Horos^®^ Public license, versión 3 (LGPL-3.0)*]. In addition, the bone reconstruction images were reviewed in the lung window (WL: −500, WW: 1400) in OsiriX to assess the visualization of the TM. The review was conducted by a first-year resident (VAN) and a board-certified radiologist (RS).

For each ear, the CT findings were documented based on previous publications (8, 9) and included the following parameters: normal or thickening of the external ear canal walls (classified as mild, moderate, or marked), external wall contrast enhancement (normal or abnormal), external ear canal mineralization (grading was considered depending on the percentage of the canal length affected: mild: 30%, moderate: 30–60%, or severe: >60%), and the presence (yes or no) and the volume (mild if it occupies less than 30% of the canal, moderate between 30 and 60%, and marked >60%) of intraluminal material within the external ear canal. The stenosis of the external ear canal was classified as mild if the maximum concentric narrowing was <30%, moderate between 30 and 60%, and severe >60%; thickening and/or lysis of the tympanic bulla walls (mild, moderate, or marked); presence (yes or no) and volume (mild if the luminal material occupied less than 30% of the cavity, moderate between 30 and 60%, and marked >60% of the cavity) of intraluminal material within the tympanic cavity; visualization of the tympanic membrane (yes or no); and the presence of regional lymphadenomegaly affecting the mandibular or retropharyngeal lymph nodes (yes or no). The authors considered the presence of irregular mineral opacities within the tympanic cavity (hyperostotic spicules) as an incidental finding.

For the CT canalography studies, the following parameters were assessed: the presence of contrast medium in the external ear canal (yes or no) and its appearance (heterogeneous or homogeneous) and the presence of the contrast medium in the tympanic cavity (yes or no) and its appearance (heterogeneous or homogeneous). The volume of contrast in the tympanic cavity was further classified as mild (if the luminal material occupied less than 30% of the cavity), moderate (between 30 and 60% of the cavity), or marked (>60% of the cavity). The authors defined a ruptured TM as the presence of a contrast medium within the tympanic cavity.

## 3 Results

A total of 11 dogs with chronic external and/or middle ear disease were included in the study. Among them, 5 dogs presented with bilateral symptoms and 6 dogs had unilateral symptoms, giving a total of 16 ears. CT canalography was performed on all 16 clinically affected ears. The volume of contrast used in the CT canalography depended on patient size; however, in general, the authors applied up to 3 mL of contrast solution, or a lower volume if the patient’s external ear canal was filled and overflowed. The procedure was well tolerated by all the dogs, and no short-term or long-term adverse reactions were observed or reported by the owners.

Ages ranged from 1 to 12 years (median 6.9 years); six breeds were represented: German Shepherd (*n* = 3), Crossbreed (*n* = 2), Golden Retriever (*n* = 2), French Bulldog (*n* = 2), Labrador Retriever (*n* = 1), and West Highland White Terrier (*n* = 1). There were seven neutered females, three entire males, and one neutered male.

### 3.1 Clinical presentation and otoscopic findings

Both ears from all patients were examined otoscopically using two otoscopic examination methods. In clinically affected ears, both otoscopic examinations of the external ear canal were abnormal in 15 of the 16 (94%) ears. The TM was visible in 1 of the 16 (6%) using conventional otoscopy and in 6 of the 16 (38%) ears using video-otoscopy, with one TM visible in both conventional and video-otoscopic examinations. The TM was considered ruptured in 2 of the 16 (13%) ears and visible but not ruptured in 4 of the 16 (25%) ears with video-otoscopy. In the remainder of the examinations of clinically affected ears, in 10 of the 16 (62%) ears, the TM was not adequately visualized with video-otoscopy (see Table 1).

**Table 1:** Otoscopic examination result.

External ear canal appearance	Abnormal with conventional otoscopy	15/16 (94%)
	Abnormal with video-otoscopy	15/16 (94%)
TM visibility	Visualized with conventional otoscopy	1/16 (6%)
	Visualized with video-otoscopy	6/16 (38%)
TM appearance (based on video-otoscopy)	Intact	4/16 (25%)
	Ruptured	2/16 (13%)
	Not seen	10/16 (63%)

TM, Tympanic membrane.

### 3.2 CT imaging findings

The imaging features identified in the CT studies are summarized in Table 2. Of the 16 clinically affected ears, 3 showed no abnormalities in the external ear canal. Additionally, 9 of the 16 ears had no abnormalities identified in the tympanic bulla. The TM was visualized in 4 of the 16 (25%) ears and identified in the bone or lung algorithms. Five patients (5 out of 11) had mild regional lymphadenomegaly, which was unilateral in two patients and bilateral in three.

**Table 2:** Results of CT imaging findings.

External ear canal	Thickening	Normal	6 (38%)
		Mild	2 (7%)
		Moderate	6 (38%)
		Marked	2 (7%)
	Mineralization	Normal	5 (31%)
		Mild	4 (25%)
		Moderate	7 (44%)
		Marked	0 (0%)
	Stenosis	Normal	6 (38%)
		Mild	2 (7%)
		Moderate	6 (38%)
		Marked	2 (7%)
	Volume of intraluminal material	No material	8 (50%)
		Mild	6 (38%)
		Moderate	2 (7%)
		Marked	0 (0%)
Tympanic bulla	Thickening	Normal	13 (81%)
		Mild	3 (19%)
		Moderate	0 (0%)
		Marked	0 (0%)
	Thinning/osteolysis	Normal	15 (94%)
		Mild	0 (0%)
		Moderate	1 (6%)
		Marked	0 (0%)
	Distension	Normal	16 (100%)
		Mild	0 (0%)
		Moderate	0 (0%)
		Marked	0 (0%)
	Volume of intraluminal material	No material	10^a^ (62%)
		Mild	0 (0%)
		Moderate	2 (7%)
		Marked	4 (25%)
	Tympanic membrane visualization	Yes	4 (25%)
		No	12 (75%)
Other	Ipsilateral regional lymphadenomegaly	Yes	4 (25%)
		No	12 (75%)

a Presence of hyperostotic tympanic bone spicules in three tympanic cavities.

### 3.3 CT canalography imaging findings

**Figure 2:**
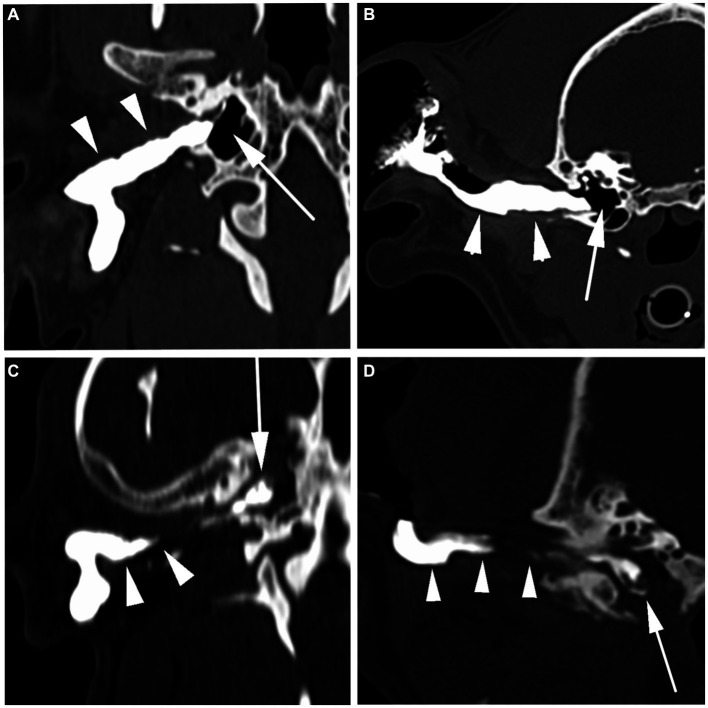
Positive contrast CT canalography images. Positive contrast CT canalography in the dorsal **(A,C)** and transverse **(B,D)** planes. In images **(A,B)**, the contrast homogeneously fills the horizontal segment of the external ear canal, and it ends at the level of the tympanic membrane (arrowheads). There is no contrast within the tympanic cavity, suggesting the tympanic membrane is intact (arrow). Images **(C,D)** show the contrast that heterogeneously fills the horizontal external ear canal, which is moderately narrowed (arrowheads). A moderate volume of contrast that heterogeneously fills the tympanic cavity, suggesting a rupture of the tympanic membrane (arrow).

All evaluators agreed that all the CT canalography images were of diagnostic quality, with no evidence of high-density streaks and blooming artifacts. In all CT canalography studies, the contrast was present within the lumen of the horizontal external ear canal in all cases. In 4 of the 16 (25%) ears, the contrast was also noted within the lumen of the ear canal. The contrast filling of the lumen of the external ear canal was homogeneous in 9 of the 16 (56%) ears (Figures 2A,B) and heterogeneous in 7 of the 16 (44%) ears (see Table 3).

**Table 3:** Results of positive contrast CT canalography imaging findings.

External ear canal	Contrast in external ear canal	Present	Not present	–
		16/16 (100%)	0/16 (0%)	–
	Contrast appearance	Homogeneous	Heterogeneous	–
		9/16 (56%)	7/16 (44%)	–
Tympanic bulla	Contrast in tympanic cavity	Yes (Positive)	No (Negative)	–
		7/16 (44%)	9/16 (56%)	–
	Volume of contrast	Mild	Moderate	Marked
		2/7 (29%)	5/7 (71%)	0/7 (0%)
	Contrast appearance	Homogeneous	Heterogeneous	–
		2/7 (29%)	5/7 (71%)	–

Confirming a ruptured TM, the presence of positive contrast medium within the tympanic cavity was identified in 7 of the 16 (44%) ears, being mild in 2 of the 16 (13%) and moderate in 5 of the 16 (31%) ears (Figures 2C,D). Of the seven positive CT canalography studies, two were identified by video-otoscopy as having a ruptured TM, while the remaining five had inadequate visualization of the TM. Additionally, two of these seven positive studies showed no CT abnormalities of the tympanic bulla, whereas the remaining five had moderate to marked volumes of intraluminal material in the tympanic cavity, with two also exhibiting bulla wall thickening.

On the other hand, of the nine negative CT canalography studies, four were found to have an intact TM with video-otoscopy, while the remaining five had inadequate TM visualization. None of the negative CT canalography studies were determined to have a ruptured TM with video-otoscopy. Among these negative CT canalography studies, only one had shown a moderate amount of intraluminal material in the tympanic cavity on CT imaging, while the tympanic membrane appeared intact on video-otoscopy. The remaining eight negative CT canalography studies showed no CT abnormalities of the tympanic bulla.

## 4 Discussion

In veterinary medicine, assessing the auditory system can be challenging due to the anatomical conformation of the external ear canal, more evident in some breeds, such as brachycephalics (3, 4). The external ear canal is generally long, curved, and funnel-shaped, and it is divided into vertical and horizontal segments. The ending of the external ear canal is delineated by the TM, which separates the external ear canal from the middle ear (2).

Otitis media is often caused by an extension of external ear disease (1). The otoscopic examination has routinely been the method of choice for evaluation of the TM, with video-otoscopy being the gold standard otoscopic technique (5). Nevertheless, the presence of a stenotic ear canal and/or intraluminal secretions and debris can make the visualization of the TM very difficult otoscopically. The inability to identify the TM during the otoscopic examination, via conventional otoscopy or video-otoscopy, indicates the presence of external ear canal disease and/or an underlying OM (6). In the present study, 15 of the 16 (94%) clinically affected ears were abnormal otoscopically, and in 10 of the 16 (63%) clinically affected ears, the tympanic membrane could not be visualized otoscopically at its normal location (with either otoscopic technique), precluding a diagnosis of TM damage.

The most common CT findings in patients with otitis externa and/or otitis media have already been published (7, 10, 13–16, 21, 22). In our results, we observed a similar CT to those published as well as regional lymphadenomegaly associated with otitis externa and/or otitis media in 8 of the 22 (36%) ears. Mineralization of the external ear canal was a frequent finding in both clinically affected and non-clinically affected ears. In one previous study, mineralization of the external ear canal was not always associated with external ear disease (16). In 3 of the 16 ears, hyperostotic spicules within the tympanic cavity were identified (17).

The absence of CT abnormalities in the bullae does not rule out OM and ruptured TM. In two of our cases, there was no suggestion of otitis media in the standard CT examination, but a moderate volume of positive contrast was present in the tympanic cavity in the CT canalography study, confirming a ruptured TM.

In human and veterinary medicine, CT and MRI are the imaging modalities of choice to assess the external, middle, and inner ear. CT provides a superior evaluation of all anatomical bony structures by avoiding superimposition. It allows for better assessment of subtle bony alterations using a sharp kernel and is more sensitive than radiography in detecting the material presence in the tympanic cavity. In addition, the availability, cost, and shorter scanning time make this modality the gold standard to evaluate the ears when compared with MRI (8, 14, 15). CT can identify the TM in some but not all dogs. The best plane reported for the visualization of the TM is the dorsal plane; however, its visualization strongly depends on the window selection. In this study, the TM was identified in only 4 of the 16 (25%) ears using standard CT. In the authors’ opinion, the optimal window to identify the TM is the lung window using a transverse plane. In CT, the TM could be effaced by material within the external and/or middle ear, limiting its visualization.

In addition to CT, MRI is sometimes used to assess the structures of the external and middle ears in dogs (6, 23). Intraluminal material in the horizontal ear canal can create a sharp outline at the level of the TM if the tympanic cavity is air-filled. This might misleadingly suggest that the TM is intact. However, a follow-up otoscopy has shown that the TM is ruptured in some cases. Therefore, MRI studies are not reliable in assessing the integrity of the TM (7).

The positive contrast ear canalography technique to evaluate the TM in radiographs was first described in 1973 (18). However, the first study to evaluate the sensitivity and usefulness of the technique was published in 1998 (11). This last study used radiographic evidence of contrast in the tympanic cavity to diagnose TM rupture, and it showed better accuracy in diagnosing a disruption of the TM in comparison with the conventional otoscopic examination. In these studies (11, 18), there were no reported complications secondary to the presence of positive contrast within the tympanic cavity.

The main aim of the present study was to describe the technique of positive contrast CT canalography. The authors used a similar protocol in the study that was utilized previously for radiographic assessment (8). The appearance of the contrast within the external ear canal and tympanic cavity, considered secondary to mixing the contrast medium with pre-existing intraluminal material or residual fluid from ear canal lavage, was not always linked to the presence of intraluminal material in the external ear canal. In the tympanic cavity, the varied filling was associated with intraluminal material in four of the five ears. Despite the irregular contrast filling, a CT canalography result could still be obtained. The images were reviewed using a DICOM viewer in the bone window reconstruction to reduce the potential high-density streak and blooming emanating artifacts (19) caused by the concentration and volume of contrast used for the CT canalography.

The second aim of the present study was to determine whether CT canalography allowed the identification of rupture of the TM. In our study, the contrast was observed within the tympanic cavity in 7 of the 16 (44%) ears, classified as a “positive CT canalography result,” indicating a ruptured TM. Among the positive CT canalography studies, TM rupture was identified in two of the seven (29%) ears during the video-otoscopic examination. However, in five of the seven (71%) ears, TM rupture went unnoticed during the video-otoscopic examination due to limitations in TM visualization. Therefore, CT canalography has the potential to be a more sensitive indicator of TM rupture than video-otoscopy. The authors have considered that the TM rupture may have been iatrogenic during the procedure or ear canal flushing. However, this is considered unlikely due to the low volume of fluid introduced, low rate of administration, use of a blunt polyurethane catheter for the introduction of contrast and flushing materials, and gentle manipulation and massage of the ear during the procedure. While iatrogenic contamination of the middle ear by external ear canal material has been reported in cases of ear canal flushing and subsequent myringotomy, this is presumed to occur through a myringotomy defect in the TM, which is not present in the current study (20). In all cases, there were no reported complications after the CT canalography by the owners.

In 9 of the 16 (56%) cases, there was no evidence of contrast within the tympanic cavity, classified as a “negative CT canalography result.” In 4 of the 9 (44%) negative CT canalography studies, the TM was considered intact during the video-otoscopic examination. In the remaining negative cases, the TM was not visible during the video-otoscopic examination in 5 of the 9 (56%) studies. It is considered possible that the presence of discharge in the external ear canal or severe stenosis could obstruct the flow of contrast preventing it from passing through a TM rupture, resulting in a false negative result. This has been minimized by flushing and cleaning the ear canal before the CT canalography, diluting the contrast to make it less viscous, and gently massaging the ear canal after the introduction of contrast to distribute the solution as much as possible. This potential limitation of canalographic TM assessment is shared by the current gold standard of video-otoscopy.

Additional limitations in this study include several other factors. The first was the low number of cases (*n* = 16 ears) assessed with CT canalography, which reduced the power of the results. Evaluation of other benefits of the positive contrast CT canalography, for example, to detect para-aural abscess or to delineate intraluminal structures (adenomatous polyps or neoplasia, among others), was considered beyond the scope of the manuscript. Further investigations are required to clarify the usefulness of this contrast technique in diagnosing other auditory pathologies.

In conclusion, CT canalography is considered a useful and safe complementary contrast study technique to evaluate the integrity of the tympanic membrane in dogs. The presence of a contrast medium within the tympanic cavity suggests a ruptured tympanic membrane. Positive CT canalography studies may be seen in patients whose tympanic membrane cannot be assessed with video-otoscopy and in patients with no CT abnormalities of the tympanic bulla. The absence of contrast within the tympanic cavity does not rule out the possibility of tympanic membrane rupture. The authors recommend the addition of this contrast study technique to the normal CT protocol in patients when there is a suspicion of middle ear disease and where video-otoscopy is not available.

## Data availability statement

The original contributions presented in the study are included in the article/Supplementary material, further inquiries can be directed to the corresponding author.

## Ethics statement

Ethical approval was not required for the studies involving animals in accordance with the local legislation and institutional requirements because in this study, we utilized an imaging technique that has previously been described in radiographs, and its safety profile has been well-established in clinical practice. The study was conducted at a private hospital, where patient care and safety are paramount. As such, we did not seek specific ethical approval for this research, as the imaging protocol and procedures closely followed established standards of care, and there was no deviation from routine clinical practice. Patient confidentiality and data protection were maintained in accordance with applicable privacy regulations. Written informed consent was obtained from the owners for the participation of their animals in this study.

## Author contributions

VA-N: Conceptualization, Data curation, Formal analysis, Supervision, Visualization, Writing – original draft. MP: Conceptualization, Formal analysis, Supervision, Visualization, Writing – review & editing. RR: Data curation, Writing – review & editing. RS: Conceptualization, Data curation, Supervision, Validation, Visualization, Writing – review & editing.
